# Prevalence and factors associated with overweight and obesity among rural and urban women in Burkina Faso

**DOI:** 10.11604/pamj.2019.34.199.20250

**Published:** 2019-12-16

**Authors:** Jeoffray Diendéré, Jean Kaboré, Jérôme Winbetourefa Somé, Gauthier Tougri, Augustin Nawidimbasba Zeba, Halidou Tinto

**Affiliations:** 1 Research Institute for Health Sciences, Bobo-Dioulasso, Burkina Faso; 2 Centre Muraz, Bobo-Dioulasso, Burkina Faso; 3 Ministry of Health, Ouagadougou, Burkina Faso; 4 Clinical Research Unit, Research Institute for Health Sciences, Nanoro, Burkina Faso

**Keywords:** Women, overweight, obesity, prevalence, associated factors, urbanization, Burkina Faso

## Abstract

**Introduction:**

Low- and middle-income countries, including Burkina Faso, are facing increasing urbanization with health challenges related to nutrition transition that impact body weight change. This study reported the prevalence and factors associated with overweight/obesity among women living in rural and urban Burkina Faso.

**Methods:**

We conducted a secondary analysis using data from the Burkina Faso 2013 WHO STEPwise survey. Data included socio-demographic, clinical (anthropometric, systolic/diastolic blood pressure (SBP/DBP), oral/dental symptoms), biological (total and high-density lipoprotein cholesterol and fasting blood sugar), and alcohol and tobacco consumption data. A total of 2191 participants with complete data were considered in the analysis. We categorized the 13 Burkinabe regions by urbanization rate quartiles. We then performed Student's t, chi-squared, and Fisher's exact tests and backward stepwise regressions.

**Results:**

The overall prevalence of overweight/obesity was 19.6% (13.1% and 44% in rural and urban women respectively, p=0.0001). Common factors positively associated with overweight/obesity in both rural and urban women were being a resident of a region in the highest urbanization rate quartile, having a high level of total cholesterol (alone or via an interaction with age) and having a high DBP. In urban women only, overweight/obesity was also associated with a high SBP.

**Conclusion:**

The prevalence of overweight/obesity in urban women in Burkina was among the highest levels in urban sub-Saharan Africa and roughly mimicked the urbanization profile of the country. In overweight/obesity conditions, cardiovascular concerns, such as increase in total cholesterol and blood pressure, were objective, and the blood pressure increase was more severe in urban women than in rural women.

## Introduction

In low- and middle-income countries (LMICs), the nutrition transition process is accompanied by body weight changes [1,2]. Between 1980 and 2008, the weight gain (in body mass index (BMI), kg/m^²^) per decade was estimated at 0.6 (95% CI: 0.0-1.1) and 0.9 (95% CI: 0.4-1.3) in West African men and women aged >20 years, respectively [3]. Overweight/obesity is known to be associated with increased cardiovascular risk [4,5]. During the same period (1980-2008), the increase in fasting blood sugar (mmol/l) per decade was estimated at 0,05 (95% CI: -0.15-0.24) in men versus 0.13 (95% CI: -0.07-0.34) in women in sub-Saharan Africa (SSA) [6]. There was also a gradual shift in the prevalence of individuals with high blood pressure (HBP) in SSA [7], and between 1994 and 2003, blood pressure levels significantly increased in rural Cameroon (for women: +18.2 mmHg for systolic blood pressure (SBP) and +11.9 mmHg for diastolic blood pressure (DBP), and for men: +18.8 mmHg for SBP and +11.6 mmHg for DBP), as well as in urban Cameroon (for women: +8.1 mmHg for SBP and +3.3 mmHg for DBP, and for men: +6.5 mmHg, p<0.001) [8]. Urbanization seems to be implied in the nutrition transition process and health-related change. Urbanization in SSA is coupled with a fundamental change [9] in the traditional lifestyle, dietary patterns, consumption behavior and level of physical activity [10]. The female sex was severely affected by weight gain [11]. In Burkina Faso, a low-income country in SSA, the last General Population and Housing Census (in 2006) counted 14,017,262 inhabitants, with 22.7% of them living in urban areas. No previous study has reported the national prevalence of overweight/obesity and the associated factors in women living in rural and urban Burkina Faso. This study aimed to assess the prevalence of overweight/obesity and the associated factors among rural and urban women in Burkina Faso by using nationally representative data.

## Methods

We used data from the Burkina Faso first national survey conducted in 2013, based on the WHO STEPwise approach to Surveillance (STEPS) [12]. The sample size calculation and the data collection process used throughout the country have been reported in two previous papers [13,14]. Complete data from 2438 participating women were available and included data on nutritional status, biological features, and alcohol and tobacco consumption. We conducted the analysis using the data for 2191 women, after excluding those who were pregnant.

### Variables of interest

**Figure 1:**
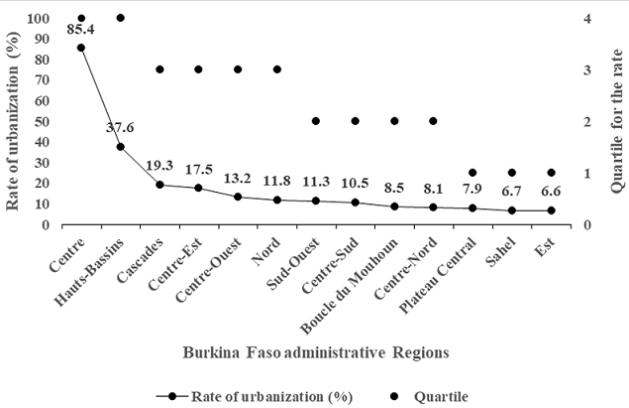
Urbanization gradient of the 13 regions of Burkina Faso based on the last (2006) General Population and Housing Census

Participant demographic information included age (ranging from 25 to 64 years), marital status (groups: i. married or cohabitating, ii. never married, iii. divorced or separated, iv. widowed), residence environment (i. urban, ii. rural), education levels (groups: i. no formal schooling, ii. primary or more), occupation (groups: i. public or private formal employment or self-employed, ii. student or homemaker or retired or unemployed or volunteer). Anthropometric data were weight (kg), height (m), BMI (weight/height², kg/m²) and waist circumference (cm). We defined overweight/obesity as BMI ≥25 kg/m². Biological data included total cholesterol (mmol/l), high density lipoprotein cholesterol (HDL-cholesterol in mmol/l, a cut-off of >1.2 mmol/l was defined as a high level) and fasting blood sugar (mmol/l, having a level ≥6.1 mmol/l was defined as high blood sugar). Blood pressure (systolic and diastolic values in mmHg) was measured three times, and we used the mean of the three values for each indicator. High blood pressure was defined as a mean value of SBP/DBP ≥140/90 mmHg or actively undergoing anti-hypertension treatment. Data on current (past month) alcohol use were recorded by the self-reported alcohol consumption technique. Current (past year) smoking tobacco use considered manufactured cigarette smoking, hand-rolled tobacco smoking, and pipe smoking. Urbanization was characterized using urbanization rates for the 13 Burkinabe regions provided by the “Institut National des Statistiques et de la Démographie (INSD)” in 2006. The urbanization rate of the region was calculated as the proportion of inhabitants living in urban areas in the region. The national mean rate of urbanization was 23.33% (minimum = 6.6%, maximum = 85.4%), and the quartile cutoffs were 8.1, 11.8 and 19.3. Four regions were included in the first quartile (Q1) or in the second quartile (Q2), three in the third quartile (Q3) and two (of the “Centre” and “Hauts-Bassins” regions) in the fourth quartile (Q4) (Figure 1). The two regions in this last quartile include the political capital Ouagadougou (located in the “Centre” region) and the economic capital Bobo-Dioulasso (in the “Hauts-Bassins” region) and were the living areas for approximately 62% of the urban dwellers of the country (46.4% for Ouagadougou, 15.4% for Bobo-Dioulasso). These two regions are densely urbanized (Figure 1).

### Statistical analysis

We used Stata statistical software for Windows (Version 12.0, College Station, StataCorp, Texas, United States) for the analysis. First, we described the socio-demographic characteristics of the participants for the whole sample and for the rural and urban subgroups. Similarly, we described the clinical, nutritional and biological features based on the urbanicity status and overweight/obesity status. After that, we performed a multivariate logistic regression on the overall sample to estimate the odds ratios of factors associated with overweight/obesity in women. We included in the final model all variables with a p-value <0.20 in the univariate analyses, except for multinomial variables, where the overall p-value in the univariate analysis was considered. A backward stepwise approach was used to construct the final model, and interactions were tested in the overall sample and subgroup analyses (residence environment). For all statistical analyses, a p-value <0.05 was considered significant.

### Ethical considerations

The protocol of the STEPS survey was approved by the Ethics Committee for Health Research of the Ministry of Health (deliberation N^o^: 2012-12092; December 05, 2012). Informed consent was systematically sought from all participants. The “Centre Muraz” (to which the authors are affiliated), a Research Institute of the Ministry of Health, has a clearance to use the database.

## Results

The overweight/obesity prevalence was 19.6% (95% CI: 18.0-21.4) in the overall sample of women and was more pronounced in urban women than in rural women (44.0% (95% CI: 39.5-48.7) and 13.1% (95% CI: 11.6-14.8)) respectively with p<0.0001 and roughly increased with the quartile of urbanization rate (p<0.0001) (Table 1). Compared to rural women, urban women had significantly higher BMI, waist circumference, SBP, DBP, glycemia and total blood cholesterol and frequently had HBP (Table 2). Compared to those without overweight/obesity, overnourished women showed a similar trend (Table 2). Using a logistic regression model, we showed in Table 3 that a rural environment (adjusted odds ratio (aOR)=0.45; 95% CI: 0.33-0.62), low quartiles of urbanization rate (global p-value < 0.0001) and no education (aOR=0.68; 95% CI: 0.50-0.91) were protective factors and independently associated with overweight/obesity in the overall sample of women (Table 3). In addition, overweight/obesity was associated with increased DBP levels (aOR=1.04; 95% CI: 1.03-1.05) and total cholesterol (aOR=1.29; 95% CI: 1.14-1.46) for the whole sample. A marginally significant and positive interaction between age and glycemia was found (p=0.089).

**Table 1:** Socio-demographic characteristics according to living area in the overall sample (n=2191)

Socio-demographic characteristics	n	Overall n=2191	Rural n=1730	Urban n=461	p
		% (95%)	% (95%)	% (95%)	
**Urbanization rate**					0.0001*
Q1	432	19.7 (18.1-21.5)	23.8 (21.8-25.9)	4.3 (2.7-6.6)	0.0001
Q2	595	27.1 (25.3-29.2)	30.8 (28.6-33.0)	13.7 (10.7-17.1)	0.0001
Q3	661	30.2 (28.3-32.1)	33.6 (31.4-35.9)	17.4 (14.0-21.1)	0.0001
Q4	503	23.0 (21.2-24.8)	11.8 (10.4-13.5)	64.6 (60.1-69.0)	0.0001
Age mean ± standard deviation*		38.7 (10.8)	37.8 (10.9)	38.64 (10.4)	0.12
**Age range (years)**					0.108*
25-34	1,009	46.1 (44.0-48.2)	46.8 (44.4-49.2)	43.4 (38.8-48.1)	0.20
35-49	774	35.3 (33.3-37.4)	34.2 (32.0-36.5)	39.5 (35.0-44.1)	0.036
>49	408	18.6 (17.0-20.3)	19.0 (17.2-21.0)	17.1 (13.8-20.9)	0.36
**Marital status**					0.0001*
Married/cohabitating	1,910	87.2 (85.7-88.6)	89.8 (88.2-91.2)	77.4 (73.4-81.2)	0.0001
Singles	281	12.8 (11.5-14.3)	10.2 (8.8-11.8)	22.6 (18.8-26.7)	0.0001
**Education level**					
No formal education	1,787	81.6 (79.9-83.2)	90.1 (88.6-91.4)	49.7 (45.0-54.3)	0.0001
Primary school or more	404	18.4 (16.8-20.1)	9.9 (8.6-11.5)	50.3 (45.6-55.0)	0.0001
**Occupation**					0.0001*
Employed/Self-employed	1,222	55.8 (53.7-57.9)	57.9 (55.6-60.3)	47.7 (43.1-52.4)	0.0001
Others	969	44.2 (42.1-46.3)	42.1 (39.7-44.4)	52.3 (47.6-56.9)	0.0001

* Overall p-value%, CI: confidence interval

**Table 2:** Nutritional, clinical and biological lifestyle features of women according to their living area and overweight and obesity status (n=2191)

Characteristics	Overall	Living area			Without or with overweight/obesity		
		Rural	Urban		Without overweight/obesity	With overweight/obesity	
	n=2191	n=1730	n = 461	p	n=1761	n=430	p
Weight, mean (sd) in kg	58.7 (12.2)	56.4 (10.2)	67.0 (15.1)	0.0001	54.3 (7.4)	76.4 (11.8)	0.0001
BMI, mean (sd) in kg/m²	22.3 (4.3)	21.5 (3.6)	25.3 (5.5)	0.0001	20.6 (2.2)	29.1 (4.2)	0.0001
Nutritional status (%)				0.0001			
Undernourishment	13.9 (12.5-15.4)	16.0 (14.3-17.8)	6.3 (4.3-8.9)		---	---	---
Normal	66.5 (64.4-68.4)	70.9 (68.7-73.1)	49.7 (45.0-54.3)		---	---	---
Overweight	13.5 (12.1-15.0)	10.1 (8.7-11.6)	26.2 (22.3-30.5)		---	---	---
Obese	6.1 (5.2-7.2)	3.0 (2.3-3.9)	17.8 (14.4-21.6)		---	---	---
Waist circumference, mean (sd) in cm	78.2 (12.7)	76.2 (11.6)	85.9 (13.8)	0.0001	74.9 (10.5)	91.8 (12.1)	0.0001
Systolic blood pressure, mean (sd) in mm Hg	120.9 (17.6)	120.1 (16.7)	124.1 (20.1)	0.0001	119.4 (16.8)	127.0 (19.1)	0.0001
Diastolic blood pressure, mean (sd) in mmHg	77.8 (10.8)	76.8 (10.0)	81.5 (12.6)	0.0001	76.6 (10.0)	82.8 (12.3)	0.0001
High blood pressure (≥140/90 mmHg), yes (%)	16.9 (15.4-18.6)	14.6 (13.0-16.4)	25.6 (23.3-31.9)	0.0001	13.5 (11.9-15.1)	31.2 (26.8-35.8)	0.0001
Blood sugar, mean (sd) in mmol/L	3.9 (1.6)	3.9 (1.5)	4.1 (1.7)	0.019	3.9 (1.5)	4.1 (1.8)	0.024
High blood sugar (> 6.1mmol/L), yes (%)	5.2 (4.2-6.1)	5.0 (4.0-6.0)	5.9 (3.7-8.0)	0.50	4.6 (3.7-5.7)	7.4 (5.2-10.3)	0.01
HDL cholesterol, mean (sd) in mmol/l	1.0 (0.5)	0.9 (0.5)	1.1 (0.5)	0.0001	1.0 (0.5)	1.0 (0.5)	0.43
Low HDL cholesterol (<1.2 mmol/l), yes (%)	79.6 (77.9-81.3)	81.2 (79.2-83.0)	74.0 (69.7-77.9)	0.001	79.6 (77.6-81.4)	80.0 (75.9-83.7)	0.80
Total cholesterol, mean (sd) in mmol/l	3.2 (0.9)	3.1 (0.8)	3.7 (1.1)	0.0001	3.1 (0.8)	3.6 (1.1)	0.0001
Presence of oral/dental symptom, yes (%)	23.2 (21.5-25.1)	24.1 (22.1-26.2)	20.0 (16.4-23.9)	0.061	24.1 (22.1-26.1)	19.8 (16.1-23.9)	0.058
Current alcohol use, yes (%)	25.07 (23.2-26.8)	24.6 (22.6-26.7)	26.5 (22.5-30.7)	0.40	25.4 (23.4-27.5)	23.3 (19.3-27.5)	0.36

Sd: standard deviation; cm: centimeter; kg: kilogram. Only one woman used the smoked tobacco. Overweight/obesity prevalence in Q1, Q2, Q3 and Q4 were respectively 10.2% (95% CI: 7.5-13.4), 15.3% (95% CI: 12.5-18.4), 12.7% (95% CI: 10.3-15.5) and 42.0% (95% CI: 37.6-46.4) overall p<0.0001

**Table 3:** Factors associated with overweight and obesity among overall women in Burkina Faso from the multivariate analysis (n=2191)

Factors	Univariate analysis			Multivariate analysis		
	cOR	95% CI	P	aOR	95% CI	p
Living environment rural vs urban	0.19	0.15-0.24	0.0001	0.45	0.33-0.62	0.0001
Urbanization rate			0.0001			0.0001
Q4	1					
Q3	0.20	0.15-0.27		0.37	0.27-0.51	
Q2	0.24	0.19-0.33		0.56	0.40-0.78	
Q1	0.16	0.11-0.22		0.36	0.24-0.55	
Age range^*^			0.257			0.2132
25-34	1					
35-49	1.34	1.06-1.70		1.52	0.76-3.04	
> 49	1.08	0.80-1.45		2.01	0.86-4.69	
Education level: No formal education vs primary or more (ref)	0.32	0.26-0.41	0.0001	0.68	0.50-0.91	0.010
Occupation: Employed/Self-employed (ref) vs others	1.08	0.88-1.34	0.460	0.90	0.71-1.14	0.370
Oral or dental symptom: presence vs absence (ref)	0.78	0.60-1.01	0.058	0.69	0.59-1.04	0.093
Current alcohol use: yes, vs no (ref)	0.89	0.70-1.14	0.361	0.87	0.66-1.15	0.340
Blood sugar (mmol/L)^*^	1.08	1.01-1.1	0.026	1.07	0.97-1.19	0.188
Total cholesterol (mmol/L)	1.69	1.51-1.89	0.0001	1.29	1.14-1.46	0.0001
Systolic blood pressure	1.02	1.01- 1.03	0.0001	1.01	> 0.99-1.02	0.116
Diastolic blood pressure	1.05	1.04-1.06	0.0001	1.04	1.03-1.05	0.0001

* p=0.0890 for interaction between age range and blood sugar

In the subgroup (rural or urban) analyses (Table 4), there was a similar association between urbanization rate and overweight/obesity, with roughly the same strength. The odds ratio for overweight/obesity was associated with increased total cholesterol levels (aOR=1.26; 95% CI: 1.07-1.48) in rural women, while the interaction effect between total cholesterol and age category was significantly associated with overweight/obesity in urban women (overall p=0.08). Regarding blood pressure, only an increase in DBP (aOR=1.02; 95% CI: >1.00-1.04; p=0.032) and not in SBP (aOR=1.01; 95% CI: >0.99-1.02; p=0.062) was significantly associated with overweight/obesity in rural women, while in urban women, the increase in both DBP (aOR=1.15; 95% CI: 1.04-1.28) and SBP (aOR=1.08; 95% CI: 1.01-1.16) were significantly associated. Current alcohol intake was significantly protective against overweight/obesity in rural areas (aOR= 0.67; 95% CI: 0.46-0.96), whereas it had a marginal association with overweight/obesity (aOR=1.59; 95% CI: 0.98-2.58; p=0.062) in urban areas. In rural areas, the presence of dental symptoms was negatively associated with overweight/obesity in rural women. Among socio-demographic factors, education and marital status and age were associated with overweight/obesity in rural and urban women, respectively. Rural women with no education were less overweight/obese than single urban women or urban women aged 34-49 years. When testing the interaction between DBP and SBP in rural and urban women, a significant association was observed in the subgroup of urban women (p=0.042).

**Table 4:** Factors associated with overweight and obesity in the subgroup of rural and urban women in Burkina Faso from the multivariate analysis

Factors	Multivariate analysis in the subgroup of rural women, n=1730			Multivariate analysis in the subgroup of rural urban women, n=461		
	aOR	95% CI	P	aOR	95% CI	P
**Urbanization rate**			0.003			0.0036
Q4	1					
Q3	0.29	0.19-0.45	0.0001	0.52	030-0.90	0.020
Q2	0.52	0.34-0.79	0.002	0.53	0.27-1.03	0.059
Q1	0.32	0.20-0.51	0.0001	0.07	0.01-0.53	0.010
Age range^*^			0.345			0.0402
25-34	1					
35-49	1.39	0.99-1.94	0.053	0.11	0.02-0.60	0.011
> 49	1.27	0.82-1.97	0.284	0.32	0.04-2.67	0.295
Marital status: singles vs married/cohabitating (ref)	0.94	0.57-1.53	0.778	0.52	0.31-0.86	0.011
Education level: no formal education vs primary or more (ref)	0.55	0.36-0.83	0.005	>1.00	0.65-1.56	0.993
Occupation: employed/Self-employed (ref) vs others	0.83	0.61-1.13	0.234	0.97	0.63-1.47	0.869
Oral or dental symptom: presence vs absence (ref)	0.61	0.42-0.89	0.010	1.05	0.62-1.79	0.847
Current alcohol use: yes, vs no (ref)	0.67	0.46-0.96	0.030	1.59	0.98-2.58	0.062
Blood sugar (mmol/L)	0.96	0.87- 1.05	0.396	1.09	0.96-1.24	0.194
Total cholesterol (mmol/L)^*^	1.26	1.07-1.48	0.005	1.07	0.78-1.46	0.682
Systolic blood pressure (mmHg)	1.01	> 0.99-1.02	0.061	1.08	1.01-1.16	0.033
Diastolic blood pressure (mmHg)	1.02	> 1.00-1.04	0.032	1.15	1.04-1.28	0.006

* In urban women, p=0.08 for interaction between age range and total cholesterol

aOR= adjusted odd ratio, CI: confident interval

## Discussion

Overall, in this study, we found that approximately one out of five women was overweight/obese, with a large difference according to living area (13% and 44% in rural and urban women, respectively).

### Overweight/obesity and cardiovascular risk factors in the overall sample

The findings in this study were consistent with the results reported in a previous study among peri-urban and urban residents in Uganda [15] and strongly suggested considering this phenomenon with regard to the environment. Indeed, living in rural areas (aOR=0.45; 95% CI: 0.33-0.62) or in regions belonging to the low quartiles of the urbanization rate (aORs=0.56 to 0.36; global-p=0.0001) were protective factors against overweight/obesity and reflected the impact of urbanization on the nutrition transition. Overweight/obesity was associated with cardiovascular disease risks in the overall sample, as it was in each subgroup of rural and urban women. The first concern was the risk of increased DBP, with an aOR=1.04 in women overall and 1.02 and 1.15 in rural and urban women, respectively (Table 3, Table 4). Only urban women with overweight/obesity had an additional risk of increased SBP, with an aOR=1.08 (Table 4). The prevalence of HBP was greater in urban women than in rural women (31.2% vs 13.5%, respectively; p=0.0001) (Table 2). The second serious cardiovascular concern was the increased total cholesterol level, with an aOR=1.29 in women overall, 1.26 in rural women and 1.73 in urban women (via an interaction in the 35-49 years subgroup). These concerns were in line with those of the WHO in LMICs and reported by some authors in SSA [16,17] as was cardiovascular risk worsening in urban areas [18].

### Rural women

The overweight/obesity prevalence in rural Burkinabe women (13.1%, 95% CI: 11.6-14.8) was similar to the results of a previous study in rural women in Ghana (15.6% for those aged 19-49 and 18.4% for those aged 40-60 years) [19,20]. A low prevalence of 6.5% was observed, but only among women aged 40-60 years in rural Burkina Faso [21]. A number of studies in rural women in several other African countries reported a higher prevalence: 21.1% in Kenya [22], 22.0% in Tanzania [23], 22.3% in Senegal [24], 28.4% in Angola [25], 29.9% in Nigeria [26], 36.7% in Zambia [16] and 58.9% in Black South Africa [27]. No education had a protective effect on overweight/obesity (aOR=0.55; 95% CI: 0.36-0.83; Table 4), and Wagner *et al.* reported that in South Africa, a high level of education was a risk factor for overweight/obesity (using a multiple linear regression analysis, exp (β)=1.10, 95% CI: 1.03-1.18 for the tertiary level referring to no formal education) [11]. Women with a high-income level may have more food availability but an unhealthy consumption behavior, resulting in nutritional insecurity. Current alcohol consumption was negatively associated with a risk of overweight/obesity in rural areas (aOR=0.68; 95% CI: 0.47-0.98), as it was in South Africa (using a multiple linear regression analysis, exp (β)=0.70; 95% CI: 0.52-0.94 for current nonproblematic consumption referring to no history of consumption) [11]. Ghrelin hormone increased the gastric emptying rate in normal humans and lowered the hunger rate [28], and alcohol has an acute inhibitory effect on human ghrelin secretion [29]. Rural areas in Burkina Faso usually have a low food availability, and rural women adjust to hunger through alcohol consumption, which suggests an undernourished situation. The presence of oral or dental symptoms was also negatively associated with the presence of overweight/obesity (aOR=0.61, 95% CI: 0.42-0.89) (Table 4). These symptoms might be related to eating difficulties/swallowing impairment resulting in insufficient food intake, causing undernourishment. The hypothesis that alcohol consumption, or the presence of oral or dental symptoms, is positively associated with undernourishment in rural women needs to be tested.

### Urban women

The overweight/obesity prevalence among urban Burkinabe women (44.0% using the WHO STEPwise database, 2013) was similar to the results reported in 2008 in women living in urban Benin using the same STEPS approach (41.3%) [30], and in Ghana (45.7%) [31], Mozambique (39.4%), Senegal (48%) [32] and Kenya (43.4% among Nairobi women) [33]. A lower prevalence (34.6%) was reported among urban Zambian women [34] and Ethiopians (12.1%, WHO STEPwise survey) [35]. The overweight/obesity trends mimicked urbanization features (prevalence of 10.2% to 42.0%, aORs=0.52 to 0.07, according to the quartile of urbanization rate, (Table 4)), and urbanized environments seemed not to favor healthy lifestyle behaviors. For instance, the use of motorcycles is common in Burkinabe cities and may increase sedentariness in citizens. In addition, women in cities might spend more time using screens (e.g. television and telephones with or without data services). These factors have been associated with obesity in women living in Accra (aOR=1.57, 95% CI: 1.20-2.07 for ownership of a television and aOR=1.55, 95% CI: 1.22-1.98 for telephone use) [36] and in Addis Ababa, where the odds ratio increased over time (aOR=1.89; 95% CI: 1.27-2.81 in 2005, and aOR=2.28; 95% CI: 1.12-4.65 in 2011, for women who watched television) [37]. The effect of these new behaviors on overweight/obesity development needs to be studied in Burkina Faso. Nonetheless, a regional and sex-specific variation in BMI distribution has been previously reported in SSA [38]. Women who used hormonal contraception (pills, intrauterine device, injections, or implants) were also more likely to be overweight than women not using any contraception method [37]. Unfortunately, we were not able to test the contraception use in the present study because this information was not collected. The subgroup of 35- to 49-year-old women was at lower risk of overweight/obesity, possibly because of the choice of our reference group (25-34 years), which might include more rural-to-urban migrants, usually considered to be at high risk for overweight/obesity [39]. Compared to married/cohabiting women, single women were less likely to be overweight/obese (aOR=0.52, 95% CI: 0.31-0.86); a similar relationship has been found in Kenyan women (when singles were the reference, aOR=1.73, 95% CI: 1.42-2.08 for married or living with a partner) [37]. Out-of-home food consumption was an emerging practice among Burkinabe citizens [40]. Those who were married/cohabiting might have a better socioeconomic position [41] and an increased number of mealtimes; they would be used to having out-of-home food for conviviality and friendship and in-home food intake to honor their partner. Current alcohol consumption was marginally associated with overweight/obesity in our results (aOR=1.59, 95% CI: 0.98-2.58; p=0.062); this was clearly shown by Asiki *et al.* in a previous study (using a bivariate analysis, an increase in BMI was associated with current nonproblematic alcohol use, β=+0.075; 95% CI: 0.003-0.148) [42]. Alcohol is a psychoactive substance, and Canton *et al.* reported that above a certain threshold, alcohol appears to stimulate appetite, in part due to elevated levels of subjective hunger [43].

## Conclusion

The prevalence of overweight/obesity in urban women in Burkina Faso was among the highest levels in urban SSA. The distribution of overweight/obesity prevalence roughly mimicked the country's urbanization profile. The lower prevalence in rural Burkina Faso might be related to the impaired food availability and more frequent undernourishment. Risk factors for cardiovascular disease, such as increased levels of blood total cholesterol or DBP with or without increased levels of SBP, were associated with overweight/obesity. In particular, the worsening of both blood pressure and total cholesterol levels in women with overweight/obesity were more severe in urban Burkina Faso. Relevant modifiable risk factors for overweight/obesity, especially those related to positive lifestyle behavior, should be promoted to reduce the prevalence and related factors for overweight/obesity.

### What is known about this topic

- Urbanization coincides with the nutrition transition process and body weight changes;
- Cardiovascular risk factors increase with body weight gain;
- The burden of cardiovascular diseases is heaviest for the low- and middle-income countries, as in sub-Saharan Africa, including Burkina Faso.

### What this study adds

- One out of five women in Burkina Faso was overweight/obese, with a large difference according to living area and urbanization rate;
- The overweight/obese level in rural areas was attenuated by undernutrition, but it was rising among urban women, reaching a level similar to that of several urban areas in sub-Saharan Africa.
- In overweight/obesity conditions, cardiovascular concerns, such as increase in total cholesterol and blood pressure, were objective and more severe in urban women.

## Competing interests

The authors declare no competing interests.

## References

[1] Popkin BM (1998). The nutrition transition and its health implications in lower-income countries. Public Health Nutr.

[2] Popkin BM, Gordon-Larsen P (2004). The nutrition transition: worldwide obesity dynamics and their determinants. Int J Obes Relat Metab Disord J Int Assoc Study Obes.

[3] Finucane MM, Stevens GA, Cowan MJ, Danaei G, Lin JK, Paciorek CJ (2011). National, regional, and global trends in body-mass index since 1980: systematic analysis of health examination surveys and epidemiological studies with 960 country-years and 9·1 million participants. Lancet Lond Engl.

[4] Zhou B, Wu Y, Yang J, Li Y, Zhang H, Zhao L (2002). Overweight is an independent risk factor for cardiovascular disease in Chinese populations. Obes Rev Off J Int Assoc Study Obes.

[5] Bastien M, Poirier P, Lemieux I, Després J-P (2014). Overview of epidemiology and contribution of obesity to cardiovascular disease. Prog Cardiovasc Dis.

[6] Danaei G, Finucane MM, Lu Y, Singh GM, Cowan MJ, Paciorek CJ (2011). National, regional, and global trends in fasting plasma glucose and diabetes prevalence since 1980: systematic analysis of health examination surveys and epidemiological studies with 370 country-years and 2·7 million participants. Lancet Lond Engl.

[7] NCD Risk Factor Collaboration (NCD-RisC) (2017). Worldwide trends in blood pressure from 1975 to 2015: a pooled analysis of 1479 population-based measurement studies with 19·1 million participants. Lancet Lond Engl.

[8] Fezeu L, Kengne AP, Balkau B, Awah PK, Mbanya JC (2010). Ten-year change in blood pressure levels and prevalence of hypertension in urban and rural Cameroon. J Epidemiol Community Health.

[9] Popkin BM (1999). Urbanization, Lifestyle Changes and the Nutrition Transition. World Dev.

[10] Delisle H, Ntandou-Bouzitou G, Agueh V, Sodjinou R, Fayomi B (2012). Urbanisation, nutrition transition and cardiometabolic risk: the Benin study. Br J Nutr.

[11] Wagner RG, Crowther NJ, Gómez-Olivé FX, Kabudula C, Kahn K, Mhembere M (2018). Sociodemographic, socioeconomic, clinical and behavioural predictors of body mass index vary by sex in rural South African adults-findings from the AWI-Gen study. Glob Health Action.

[12] Bonita R, Winkelmann R, Douglas KA, de Courten M, McQueen DV, Puska P (2003). The WHO Stepwise Approach to Surveillance (Steps) of Non-Communicable Disease Risk Factors. Global Behavioral Risk Factor Surveillance.

[13] Soubeiga JK, Millogo T, Bicaba BW, Doulougou B, Kouanda S (2017). Prevalence and factors associated with hypertension in Burkina Faso: a countrywide cross-sectional study. BMC Public Health.

[14] Millogo T, Bicaba BW, Soubeiga JK, Dabiré E, Médah I, Kouanda S (2018). Diabetes and abnormal glucose regulation in the adult population of Burkina Faso: prevalence and predictors. BMC Public Health.

[15] Kirunda BE, Fadnes LT, Wamani H, Van den Broeck J, Tylleskär T (2015). Population-based survey of overweight and obesity and the associated factors in peri-urban and rural Eastern Uganda. BMC Public Health.

[16] Tateyama Y, Techasrivichien T, Musumari PM, Suguimoto SP, Zulu R, Macwan'gi M (2018). Obesity matters but is not perceived: A cross-sectional study on cardiovascular disease risk factors among a population-based probability sample in rural Zambia. PloS One.

[17] Duda RB, Kim MP, Darko R, Adanu RMK, Seffah J, Anarfi JK (2007). Results of the Women's Health Study of Accra: assessment of blood pressure in urban women. Int J Cardiol.

[18] Fezeu L, Balkau B, Sobngwi E, Kengne A-P, Vol S, Ducimetiere P (2010). Waist circumference and obesity-related abnormalities in French and Cameroonian adults: the role of urbanization and ethnicity. Int J Obes 2005.

[19] Doku DT, Neupane S (2015). Double burden of malnutrition: increasing overweight and obesity and stall underweight trends among Ghanaian women. BMC Public Health.

[20] Nonterah EA, Debpuur C, Agongo G, Amenga-Etego L, Crowther NJ, Ramsay M (2018). Socio-demographic and behavioural determinants of body mass index among an adult population in rural Northern Ghana: the AWI-Gen study. Glob Health Action.

[21] Boua RP, Sorgho H, Rouamba T, Nakanabo Diallo S, Bognini JD, Konkobo SZ (2018). Gender differences in sociodemographic and behavioural factors associated with BMI in an adult population in rural Burkina Faso - an AWI-Gen sub-study. Glob Health Action.

[22] Keino S, Plasqui G, van den Borne B (2014). Household food insecurity access: a predictor of overweight and underweight among Kenyan women. Agric Food Secur.

[23] Keding GB, Msuya JM, Maass BL, Krawinkel MB (2013). Obesity as a public health problem among adult women in rural Tanzania. Glob Health Sci Pract.

[24] Macia E, Cohen E, Boetsch G, Boetsch L, Cohen E, Duboz P (2017). Prevalence of obesity and body size perceptions in urban and rural Senegal: new insight on the epidemiological transition in West Africa. Cardiovasc J Afr.

[25] Pedro JM, Brito M, Barros H (2018). Gender and socio-demographic distribution of body mass index: The nutrition transition in an adult Angolan community. J Public Health Afr.

[26] Sola AO, Steven AO, Kayode JA, Olayinka AO (2011). Underweight, overweight and obesity in adults Nigerians living in rural and urban communities of Benue State. Ann Afr Med.

[27] Alberts M, Urdal P, Steyn K, Stensvold I, Tverdal A, Nel JH (2005). Prevalence of cardiovascular diseases and associated risk factors in a rural black population of South Africa. Eur J Cardiovasc Prev Rehabil.

[28] Levin F, Edholm T, Schmidt PT, Grybäck P, Jacobsson H, Degerblad M (2006). Ghrelin Stimulates Gastric Emptying and Hunger in Normal-Weight Humans. J Clin Endocrinol Metab.

[29] Calissendorff J, Danielsson O, Brismar K, Röjdmark S (2005). Inhibitory effect of alcohol on ghrelin secretion in normal man. Eur J Endocrinol.

[30] Gbary AR, Kpozehouen A, Houehanou YC, Djrolo F, Amoussou MP, Tchabi Y (2014). Prevalence and risk factors of overweight and obesity: findings from a cross-sectional community-based survey in Benin. Glob Epidemic Obes.

[31] Pereko KK, Setorglo J, Owusu WB, Tiweh JM, Achampong EK (2013). Overnutrition and associated factors among adults aged 20 years and above in fishing communities in the urban Cape Coast Metropolis, Ghana. Public Health Nutr.

[32] Macia E, Gueye L, Duboz P (2016). Hypertension and Obesity in Dakar, Senegal. PLOS ONE.

[33] Ettarh R, Van de Vijver S, Oti S, Kyobutungi C (2013). Overweight, Obesity, and Perception of Body Image Among Slum Residents in Nairobi, Kenya, 2008-2009. Prev Chronic Dis.

[34] Amugsi DA, Dimbuene ZT, Mberu B, Muthuri S, Ezeh AC (2017). Prevalence and time trends in overweight and obesity among urban women: an analysis of demographic and health surveys data from 24 African countries, 1991-2014. BMJ Open.

[35] Abrha S, Shiferaw S, Ahmed KY (2016). Overweight and obesity and its socio-demographic correlates among urban Ethiopian women: evidence from the 2011 EDHS. BMC Public Health.

[36] Tebekaw Y, Teller C, Colón-Ramos U (2014). The burden of underweight and overweight among women in Addis Ababa, Ethiopia. BMC Public Health.

[37] Mkuu RS, Epnere K, Chowdhury MAB (2018). Prevalence and Predictors of Overweight and Obesity Among Kenyan Women. Prev Chronic Dis.

[38] Ramsay M, Crowther NJ, Agongo G, Ali SA, Asiki G, Boua RP (2018). Regional and sex-specific variation in BMI distribution in four sub-Saharan African countries: The H3Africa AWI-Gen study. Glob Health Action.

[39] Peters R, Amugsi DA, Mberu B, Ensor T, Hill AJ, Newell JN (2019). Nutrition transition, overweight and obesity among rural-to-urban migrant women in Kenya. Public Health Nutr.

[40] Ouédraogo HZ, Fournet F, Martin-Prével Y, Gary J, Henry MC, Salem G (2008). Socio-spatial disparities of obesity among adults in the urban setting of Ouagadougou, Burkina Faso. Public Health Nutr.

[41] Addo J, Smeeth L, Leon DA (2009). Obesity in urban civil servants in Ghana: Association with pre-adult wealth and adult socio-economic status. Public Health.

[42] Asiki G, Mohamed SF, Wambui D, Wainana C, Muthuri S, Ramsay M (2018). Sociodemographic and behavioural factors associated with body mass index among men and women in Nairobi slums: AWI-Gen Project. Glob Health Action.

[43] Caton SJ, Ball M, Ahern A, Hetherington MM (2004). Dose-dependent effects of alcohol on appetite and food intake. Physiol Behav.

